# Marine-Derived Polysaccharide Nanofibers for Wound Healing: Mechanistic Rationale, Biofabrication Strategies, and Translational Barriers

**DOI:** 10.3390/ph19071081

**Published:** 2026-07-13

**Authors:** Vaishali Sharma, Devesh Kumar, Ankit Awasthi, Mohit Kumar, Dinesh Kumar, Neeraj Choudhary, Emad M. Abdallah

**Affiliations:** 1 Chitkara College of Pharmacy, Chitkara University, Rajpura 140401, Punjab, India; vaishalisharma5829@gmail.com (V.S.); deveshkumar96755@gmail.com (D.K.); awasthiankit458@gmail.com (A.A.); 2 GNA School of Pharmacy, GNA University, Phagwara 144401, Punjab, India; dineshpotlia123@gmail.com (D.K.); dr.neerajchoudhary@gmail.com (N.C.); 3 Department of Biology, College of Science, Qassim University, Buraydah 51452, Saudi Arabia

**Keywords:** marine polysaccharides, nanofibers, wound healing, inflammation, drug development, chronic wounds, immunomodulation, translational barriers

## Abstract

Chronic wounds are associated with long-standing inflammation, impaired angiogenesis, oxidative stress, microbial load and defective remodelling of the extracellular matrix, impairing tissue repair. Conventional dressings offer protection and moisture regulation but do not sufficiently address the biological failures. Electrospun nanofibrous wound dressings offer a more active regenerative platform due to their architecture, which resembles the extracellular matrix, allowing cell adhesion and migration and facilitating the localised delivery of therapeutic agents. Marine-derived polysaccharides, such as alginate, chitosan, carrageenan, fucoidan, glycosaminoglycans, and ulvan, are particularly attractive in this area due to their biocompatibility, biodegradability, sustainability, and intrinsic haemostatic, antimicrobial, anti-inflammatory, antioxidant, and immunomodulatory properties. This review critically discusses the mechanistic and translational relevance of marine polysaccharide-based nanofibres in wound healing with a focus on inflammation resolution, polarisation of macrophages, responses of keratinocytes and fibroblasts, angiogenesis, collagen deposition, redox balance and matrix remodelling. Biofabrication strategies, especially electrospinning and related nanofibre-forming strategies, are reviewed from the aspects of scaffold architecture, drug-loading capacity, controlled release, and wound microenvironment modulation. The review also discusses current shortcomings such as heterogeneity in the composition of marine polymers, mechanical fragility, sterilisation and storage issues, scalability, regulatory uncertainty and limited translation from preclinical models to clinical evidence. Overall, marine-derived polysaccharide nanofibers are a promising class of multifunctional wound dressings, but their clinical translation needs stronger standardisation, comparative in vivo evidence, safety validation and manufacturable designs.

## 1. Introduction

Skin serves as the body’s primary protective barrier against environmental, physical, and microbial insults. Any disruption in skin integrity due to trauma, burns, surgery, or chronic diseases can compromise tissue homeostasis and immune defense, leading to delayed healing and increased morbidity [1,2]. Wounds are generally categorised as acute or chronic depending on the duration of healing. Acute wounds typically heal within three weeks, whereas chronic wounds persist beyond three months due to impaired inflammation resolution, infection, oxidative stress, and defective extracellular matrix (ECM) remodelling [3,4]. Chronic wounds such as diabetic foot ulcers, venous ulcers, and pressure ulcers affect millions of people worldwide, and their prevalence is steadily increasing because of population ageing, diabetes, obesity, and cardiovascular disorders. It is estimated that chronic wounds affect nearly 1–2% of the global population, imposing a major socioeconomic burden through prolonged hospitalisation, increased healthcare expenditure, and reduced quality of life [5,6].

Conventional wound dressings mainly provide passive coverage and moisture retention but often fail to actively regulate the complex biological environment of chronic wounds, including persistent inflammation, microbial colonisation, and impaired tissue regeneration [7]. In recent years, nanofibrous wound dressings have emerged as advanced alternatives due to their extracellular matrix-like fibrous architecture, high porosity, large surface-area-to-volume ratio, and ability to support cell adhesion, migration, oxygen exchange, and exudate management [8]. Additionally, nanofibers can function as localised delivery systems for therapeutic agents such as antimicrobials, antioxidants, anti-inflammatory compounds, and growth factors, transforming wound dressings from passive barriers into bioactive regenerative platforms.

Among the biomaterials explored for nanofiber fabrication, marine-derived polysaccharides have gained particular attention for their abundance, sustainability, biodegradability, and intrinsic therapeutic properties. Polysaccharides such as alginate, chitosan, carrageenan, fucoidan, glycosaminoglycans, and ulvan possess hemostatic, antimicrobial, antioxidant, anti-inflammatory, and immunomodulatory properties that directly support wound repair [9,10]. When fabricated into nanofibrous scaffolds using electrospinning and related techniques, these materials can mimic the native ECM while modulating critical wound-healing pathways, including macrophage polarisation, angiogenesis, fibroblast activation, collagen deposition, and oxidative stress regulation [11,12]. This review provides a comprehensive framework that links marine-derived polysaccharide nanofibers to their molecular roles in wound healing, beyond conventional material-based discussions. It highlights how these biomaterials regulate inflammation, oxidative stress, angiogenesis, and ECM remodelling through key signaling pathways. Additionally, it critically addresses fabrication strategies, scalability, and regulatory challenges, offering a translational roadmap for next-generation wound dressings.

## 2. Methodology

This narrative review was developed through a structured literature search designed to identify relevant evidence on marine-derived polysaccharides, nanofibrous wound dressings, and wound-healing mechanisms. Searches were performed in PubMed/MEDLINE, Scopus, Web of Science, and Google Scholar using combinations of the following terms: “marine polysaccharides,” “alginate,” “chitosan,” “carrageenan,” “fucoidan,” “ulvan,” “glycosaminoglycans,” “nanofibers,” “electrospinning,” “wound healing,” “chronic wounds,” “diabetic wounds,” “angiogenesis,” “macrophage polarization,” “oxidative stress,” “extracellular matrix remodeling,” and “controlled drug delivery.” No strict lower date limit was imposed because foundational studies on wound biology, extracellular matrix remodeling, angiogenesis, and nanofiber fabrication were required to establish mechanistic context. However, priority was given to recent peer-reviewed studies, particularly those published from 1993 onward, when discussing marine polysaccharide-based nanofibers, advanced wound dressings, controlled delivery systems, and translational barriers. Older studies were retained only when they provided essential mechanistic, methodological, or historical value. Publications were prioritized when they provided experimental evidence on scaffold properties, bioactivity, wound closure, inflammation control, antimicrobial effects, angiogenesis, collagen deposition, or translational feasibility. Publications with limited methodological detail, inaccessible full text, unclear material composition, or weak relevance to marine polysaccharide-based nanofibers were excluded from detailed discussion. Because this article is a narrative review rather than a systematic review or meta-analysis, evidence was synthesized qualitatively, with emphasis on mechanistic plausibility, material performance, biological outcomes, and clinical translation.

## 3. Phases of Wound Healing

**Figure 1:**
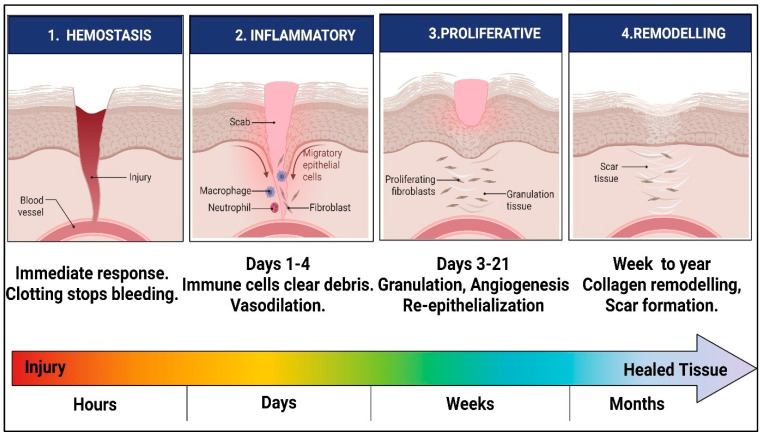
Schematic representation of the sequential and overlapping phases of normal cutaneous wound healing. The process begins with haemostasis, where platelet activation, vasoconstriction, and fibrin clot formation limit blood loss. This is followed by inflammation, during which neutrophils and macrophages remove pathogens and tissue debris. The proliferative phase involves fibroblast migration, granulation tissue formation, angiogenesis, collagen deposition, and re-epithelialization. During remodelling, extracellular matrix reorganization and collagen maturation improve tensile strength and lead to scar formation.

Wound healing encompasses various types, including primary healing (healing by first intention), delayed primary healing, secondary healing (healing by second intention), and the healing of superficial (partial-thickness) wounds. These classifications are based on the nature of the injury and the duration required for recovery [13,14]. Compromised skin integrity can disrupt cutaneous wound healing, a highly controlled and challenging process that is divided into four phases: homeostasis, inflammation, proliferation, and tissue remodelling (Figure 1) [15]. Acute wounds typically heal in a systematic and effective manner, progressing through the four overlapping phases of wound healing: haemostasis, inflammation, proliferation, and remodelling. On the contrary, chronic wounds initiate the healing process but experience extended inflammatory, proliferative, or remodelling phases, leading to tissue fibrosis and non-healing ulcers [16]. Chronic wounds generally arise as consequences of other disease processes, such as diabetic foot ulcers, pressure ulcers from spinal cord injuries, and those resulting from neurodegenerative conditions [1].

### 3.1. Haemostasis

Haemostasis is the first stage of wound healing, beginning as soon as the wound occurs and serving as the initial line of defense against excessive blood loss [17]. After tissue damage, blood vessels rapidly vasoconstrict to minimize bleeding, and platelets attach to the exposed extracellular matrix and activate [18]. The activated platelets clump together at the wound and send out several important growth factors, such as platelet-derived growth factor (PDGF), transforming growth factor-beta (TGF-β), and vascular endothelial growth factor (VEGF), that start the healing process. At the same time the coagulation pathway is also activated, causing the formation of a fibrin clot. This fibrin network not only holds the wound open but also serves as a temporary scaffold to guide the recruitment of reparative and inflammatory cells to the wound site [19].

### 3.2. Inflammation

Hemostasis is followed by the inflammatory phase, which usually begins within a few hours of injury [20]. This is an essential stage for preventing infection and removing cell debris. Neutrophils are the first of a series of immune cells to arrive at the site of a wound, and are responsible for phagocytosis and the release of antimicrobial substances, proteases, and reactive oxygen species (ROS) to destroy invading pathogens [21]. Neutrophils slowly decrease, and macrophages become the most common inflammatory cells within 48–72 h. Macrophages are integral to the orchestration of healing response through persistent phagocytosis and the release of pro-inflammatory cytokines (TNF-α, IL-1β and IL-6) and growth factors (TGF-β and VEGF) [22]. These mediators help recruit fibroblasts and endothelial cells, setting the stage for wound regeneration. To prevent tissue damage and impaired healing, it is important that the inflammatory response is controlled and does not last for too long.

### 3.3. Proliferation

The next phase of the wound is proliferative, typically beginning within 2–3 days after inflammation and lasting up to 2 weeks. Granulation tissue, angiogenesis, fibroblast proliferation, collagen deposition and re-epithelialization are features of this phase [19]. The fibroblasts infiltrate the wound bed and begin producing proteins that make up the extracellular matrix (ECM), which provides the regenerated tissue with its structure: collagen, fibronectin, and proteoglycans [23]. Meanwhile, endothelial cells begin to do their part in forming new blood vessels, providing oxygen and nutrients necessary for healing. Re-epithelialization involves the migration and proliferation of keratinocytes from the wound edges across the wound surface to repair the epidermal barrier [24]. These cellular events are regulated by growth factors, including epidermal growth factor (EGF), fibroblast growth factor (FGF), and vascular endothelial growth factor (VEGF), which are important factors in promoting tissue repair [25].

### 3.4. Remodeling

Remodeling is the last stage in wound healing and can take a few months to a year after the wound occurs [26]. In this stage, the provisional extracellular matrix created in the proliferation stage is remodelled and consolidated. Initially, the wound bed is filled with type III collagen, and over time, it is replaced by type I collagen, which has higher tensile strength and structural stability [27]. The process is closely regulated by the matrix metalloproteinases (MMPs) and their inhibitors, the tissue inhibitors of metalloproteinases (TIMPs), which govern collagen degradation and remodeling. Also, myofibroblasts contribute to wound contraction by bringing the wound edges together, thereby reducing wound size [28]. As remodeling continues, vascularity and cellularity diminish, and granulation tissue changes to scar tissue [29]. This stage increases tissue strength, but the healed tissue is only about 70–80% as strong as normal skin tissue.

## 4. Polysaccharide-Mediated Modulation of Wound Healing Pathways

Wound healing is a highly coordinated biological process involving overlapping phases of haemostasis, inflammation, proliferation, and remodelling [30]. These phases are tightly regulated by several intracellular signalling pathways that control inflammatory responses, oxidative stress, cell proliferation, migration, angiogenesis, and extracellular matrix (ECM) remodelling. Natural polysaccharides are not merely structural biomaterials but biologically active compounds capable of modulating these molecular pathways [31]. Their unique physicochemical properties, such as biodegradability, biocompatibility, mucoadhesion, and structural similarity to ECM components, enable them to interact with growth factors, cytokines, and cell-surface receptors. Through these interactions, polysaccharides regulate major signalling pathways, including Nuclear factor kappa-light-chain-enhancer of activated B cells, Nuclear factor erythroid 2-related factor 2, Phosphoinositide 3-kinase/Protein kinase B pathway, and Transforming growth factor beta signalling pathway, thereby accelerating tissue repair and restoring skin integrity [32].

### 4.1. Regulation of Inflammatory Signalling via the NF-κB Pathway

Inflammation is an essential early stage of wound healing; however, prolonged or excessive inflammation can impair tissue repair and lead to chronic wounds. The NF-κB pathway plays a central role in regulating inflammatory mediators such as TNF-α, IL-1β, IL-6, and COX-2. Natural polysaccharides have demonstrated significant anti-inflammatory effects by modulating this pathway. Chitosan has been widely reported to inhibit NF-κB activation, thereby reducing pro-inflammatory cytokine production and limiting macrophage overactivation [33]. Similarly, Fucoidan suppresses the nuclear translocation of NF-κB, thereby reducing inflammatory infiltration and improving wound closure [34]. Carrageenan also exhibits immunomodulatory properties by influencing inflammatory cytokine release. By attenuating excessive inflammation, these polysaccharides create a favourable microenvironment for progression into the proliferative phase [35].

### 4.2. Enhancement of Antioxidant Defense Through Nrf2 Signalling

Oxidative stress caused by excessive reactive oxygen species (ROS) is a major barrier to effective wound healing, particularly in diabetic and chronic wounds. The Nrf2 pathway is a key regulator of cellular antioxidant defense mechanisms, controlling the expression of heme oxygenase-1 (HO-1), superoxide dismutase (SOD), glutathione peroxidase (GPx), and catalase. Sulfated polysaccharides such as Fucoidan and Ulvan have shown potent antioxidant activity by activating Nrf2 signaling, thereby reducing ROS accumulation and protecting cells from oxidative damage [36]. Chitosan oligosaccharides also contribute to redox balance by scavenging free radicals and enhancing the activity of endogenous antioxidant enzymes. This antioxidant protection improves fibroblast viability, supports keratinocyte function, and accelerates tissue regeneration [37].

### 4.3. Promotion of Cell Proliferation and Migration Through PI3K/Akt Signalling

The proliferative phase of wound healing requires rapid fibroblast proliferation, keratinocyte migration, and granulation tissue formation [38]. The PI3K/Akt pathway is critical for these processes as it regulates cell survival, growth, and angiogenesis. Polysaccharide-based biomaterials such as Alginate and chitosan scaffolds have been shown to activate PI3K/Akt signalling, leading to enhanced fibroblast proliferation and migration [39]. This pathway also stimulates endothelial cell function, promoting blood vessel formation and nutrient supply to the wound site. Carrageenan has similarly demonstrated improved cell adhesion and migration. By stimulating PI3K/Akt signalling, these polysaccharides support rapid tissue granulation and re-epithelialization [40].

### 4.4. Extracellular Matrix Remodelling Through TGF-β/Smad Signalling

Extracellular matrix remodelling is crucial for restoring tissue structure and mechanical strength. The TGF-β/Smad pathway regulates fibroblast differentiation, collagen deposition, and scar formation. Natural polysaccharides, especially Glycosaminoglycans, structurally resemble native ECM components and directly influence TGF-β signalling [41]. Chitosan derivatives stimulate fibroblast proliferation and collagen synthesis by activating Smad proteins [14], while alginate-based dressings promote collagen maturation and the regeneration of organised tissue [42]. Hyaluronic acid, a major GAG, promotes hydration and facilitates cell migration while regulating collagen balance [43]. This modulation of ECM remodelling improves wound tensile strength and reduces abnormal scar formation.

### 4.5. Angiogenesis Stimulation via VEGF Signalling

Angiogenesis is vital for delivering oxygen and nutrients to regenerating tissue [44]. Vascular endothelial growth factor (VEGF) is one of the most important mediators of neovascularisation. Several polysaccharides enhance angiogenesis by stimulating VEGF expression. Fucoidan has shown strong pro-angiogenic activity by interacting with growth factors and promoting endothelial cell proliferation [45]. Alginate serves as an effective delivery system for VEGF and other angiogenic molecules, prolonging their release at the wound site [46]. Chitosan-based nanofibers have also demonstrated enhanced capillary formation [47]. Improved angiogenesis accelerates granulation tissue formation and enhances oxygenation of the wound microenvironment.

### 4.6. Regulation of Matrix Metalloproteinases (MMPs) and Tissue Remodelling

**Figure 2:**
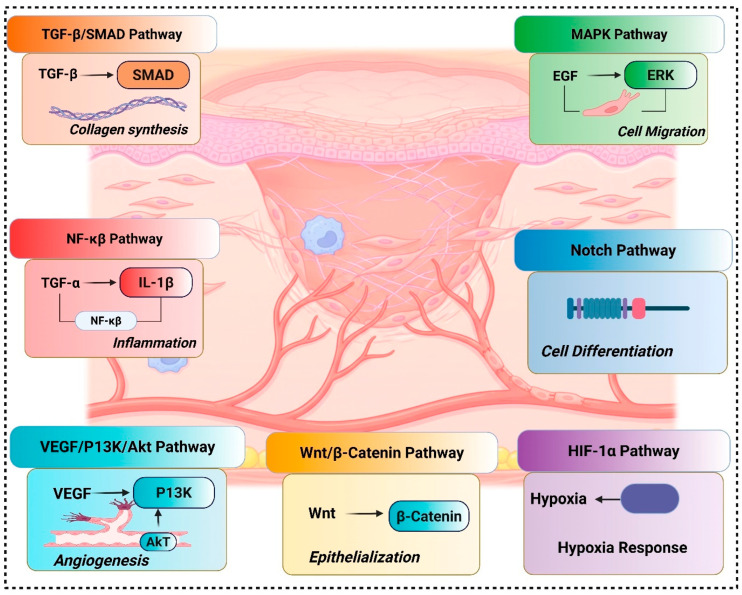
Major molecular signalling pathways involved in wound healing. The diagram summarises key regulatory axes that coordinate the resolution of inflammation, control of oxidative stress, cell migration, proliferation, angiogenesis, extracellular matrix remodelling, and immune modulation. NF-κB signalling contributes to the early production of inflammatory cytokines, whereas Nrf2/Keap1 signalling regulates antioxidant defence. PI3K/Akt, MAPK/ERK, TGF-β/Smad, Wnt/β-catenin, and HIF-1α/VEGF pathways regulate keratinocyte and fibroblast activity, endothelial responses, collagen deposition, and neovascularisation. Dysregulation of these pathways may contribute to persistent inflammation, impaired angiogenesis, excessive protease activity, delayed re-epithelialization, and chronic wound formation.

The last phase of remodelling involves a balance between matrix synthesis and degradation, largely regulated by matrix metalloproteinases (MMPs) and their inhibitors (TIMPs) [48]. Chronic wounds often exhibit excessive MMP activity, resulting in ECM breakdown and delayed healing [49]. Polysaccharides such as chitosan and GAGs have been shown to regulate MMP expression, restoring the balance between degradation and synthesis. Chitosan reduces MMP-9 activity while promoting collagen stabilization [50], whereas hyaluronic acid regulates TIMP expression to support controlled tissue remodeling [51]. This balanced ECM turnover facilitates scar maturation and improves the overall quality of healed tissue. Figure 2 shows the molecular signalling pathways of wound healing.

## 5. Nanofibers for Wound Healing

Nanofibers have emerged as a transformative platform in wound healing due to their ability to structurally and functionally mimic the native extracellular matrix (ECM) [11]. With diameters typically ranging between 50 and 500 nm, nanofibrous scaffolds provide a highly porous, interconnected network that resembles collagen fibrils in the dermal matrix [52]. This biomimetic architecture promotes cellular adhesion, proliferation, and migration, key processes required for efficient tissue regeneration. Unlike conventional wound dressings such as gauze or films, nanofibers create a moist yet breathable microenvironment that facilitates oxygen exchange while preventing excessive dehydration, thereby accelerating re-epithelialization and reducing scar formation [53]. One of the most significant advantages of nanofiber-based wound dressings is their exceptionally high surface-area-to-volume ratio, which enables efficient absorption of wound exudates and enhances interaction with surrounding tissues [11]. This structural feature also makes them ideal carriers for therapeutic agents. Bioactive compounds such as antibiotics, anti-inflammatory drugs, growth factors, metallic nanoparticles, and herbal extracts can be incorporated within the nanofibrous matrix to enable sustained and localised drug delivery [54]. Such controlled release systems are particularly valuable in chronic wounds, including diabetic ulcers and burn injuries, where prolonged inflammation and microbial colonisation delay healing. Both natural and synthetic polymers are widely employed in the fabrication of nanofibrous wound dressings. Natural polymers such as chitosan, collagen, gelatin, and alginate offer inherent biocompatibility, biodegradability, and, in some cases, antimicrobial or hemostatic properties [55]. However, their limited mechanical strength often necessitates blending with synthetic polymers. Biodegradable synthetic polymers such as Poly(lactic-co-glycolic acid), Polycaprolactone, Polyvinyl alcohol, and Polylactic acid provide improved mechanical integrity, tunable degradation rates, and enhanced structural stability [56]. Hybrid nanofibers combining natural and synthetic polymers often demonstrate synergistic benefits, integrating biological activity with mechanical robustness.

### 5.1. Methods of Preparation of Nanofibers

Nanofibers can be prepared using various top–down and bottom–up fabrication techniques, each offering distinct control over fibre diameter, morphology, porosity, and functional performance. The selection of an appropriate method depends on the polymer type, desired structural features, scalability, and intended biomedical application. Among the available approaches, electrospinning, self-assembly, phase separation, drawing, centrifugal spinning, and template synthesis are the most widely explored. These methods have been extensively investigated for the development of nanofibrous scaffolds and wound dressings due to their ability to mimic the native extracellular matrix.

#### 5.1.1. Electrospinning

**Figure 3:**
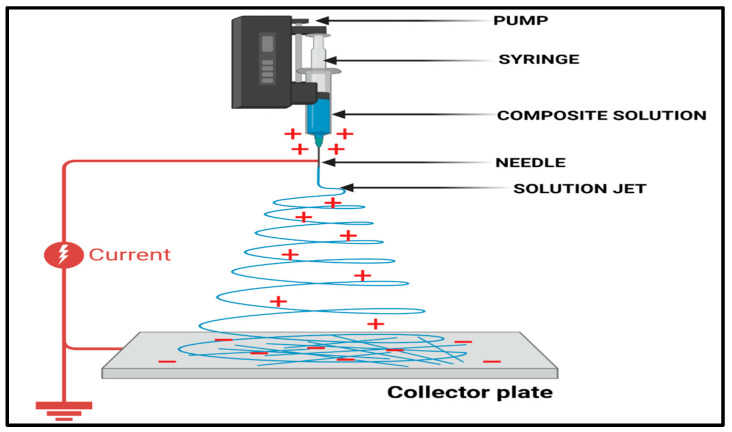
Schematic representation of the electrospinning process used to fabricate nanofibrous wound-dressing scaffolds. A polymer solution or melt is delivered through a syringe needle under a high-voltage electric field, forming a charged jet that elongates, undergoes solvent evaporation, and deposits as ultrafine fibers on a grounded collector. Key process parameters, including polymer concentration, solvent composition, applied voltage, flow rate, and tip-to-collector distance, influence fiber diameter, morphology, porosity, and drug-loading performance.

Electrospinning is the most commonly used process for preparing nanofibers due to its simplicity, scalability, and versatility in using synthetic and natural polymers. The process is performed in a high-voltage electric field on a polymer solution or melt, forming continuous ultrafine fibres that are collected on a grounded collector. Factors that may influence fibre morphology include polymer concentration, solvent system, applied voltage, flow velocity and the distance between the tip and collector [57]. Electrospun nanofibers facilitate cell adhesion, cell growth, and cell migration due to their resemblance to the fibrous network of the native extracellular matrix. Furthermore, to incorporate bioactive agents into electrospun fibres, mix, coaxial, or emulsion electrospinning could be used to introduce growth hormones, antibiotics, antioxidants and anti-inflammatory drugs (Figure 3) [58]. Zhang et al. revealed that pre-treatment of oxidative stress and inflammation by curcumin-loaded electrospun nanofibers significantly enhanced wound healing [59]. Electrospinning has a low production yield and limited control over pore size, which limits its ability to enter deeply into tissue.

#### 5.1.2. Self-Assembly

The molecules spontaneously form nanofibrous structures through non-covalent forces such as hydrogen bonding, hydrophobic interactions, and electrostatic interactions during self-assembly, a bottom-up nanofabrication method. This technique can be used to produce peptide- and protein-based nanofibers that are finely assembled at the molecular level [60]. Self-assembled nanofibers have excellent biocompatibility and bioactivity, making them attractive for tissue regeneration and wound healing. Hartgerink et al. demonstrated that peptide amphiphile nanofibers can induce angiogenesis and accelerate wound healing by mimicking ECM signalling [61]. The general application of self-assembled nanofibers is, however, hindered by their low mechanical strength, the high cost of peptides, and the complex manufacturing process.

#### 5.1.3. Phase Separation

A homogeneous polymer solution demixes to form polymer-rich and polymer-poor phases during cooling, the principle behind thermally induced phase separation (TIPS), a polymer nanofiber production procedure. The production of nanofibrous porous scaffolds is done through solvent extraction and freeze-drying [62]. Phase-separation-generated nanofibers are porous and three-dimensional, which is an advantage for nutrient diffusion and cell penetration. Ma et al. were able to prepare nanofibrous scaffolds using poly(L-lactic acid) and TIPS to demonstrate better collagen deposition and fibroblast attachment [63]. The technique, however, is often restricted to specific polymer-solvent combinations and offers inadequate control over fibre diameter and morphology compared with electrospinning.

#### 5.1.4. Drawing Technique

To form continuous nanofibers, a viscoelastic polymer droplet or melt is drawn. This method can be used in applications that require anisotropic constructions because it can be used to accurately adjust fibre alignment and diameter [64]. Low production rates and the requirement for polymers with specific viscoelastic properties limit the method, despite the uniform shape and exceptionally high mechanical integrity of pulled nanofibers. Consequently, its application in wound-healing scaffolds remains at the laboratory level.

#### 5.1.5. Centrifugal Spinning (Forcespinning)

To prepare nanofibers, centrifugal spinning (also called forcespinning) replaces electrostatic force with centrifugal force. Polymer solutions or melts are ejected out of the solvent, via small holes, at a high rotating speed, forming nanofibers [65]. This approach has several strengths over electrospinning: higher production rates, no high-voltage requirements, and greater polymer compatibility. The opportunities for large-scale production of wound dressings were described by Badrossamay et al., who developed homogeneous nanofibrous scaffolds for biomedical applications via centrifugal spinning. However, precise filling of the medication and uniform size of the fibre are not easily achieved [66].

#### 5.1.6. Template Synthesis

Template synthesis refers to the process of elaborating nanofibers within nanoporous scaffolds, such as anodic aluminium oxide membranes. Polymer deposition involves removing the template to create nanofibers of precise sizes [67]. This method is limited in wound-healing practice because it is labour-intensive and costly and cannot be applied to mass production, though it offers a high degree of control over fibre diameter and orientation.

## 6. Marine-Derived Polysaccharide-Based Nanofibers for Wound Healing Treatment

**Figure 4:**
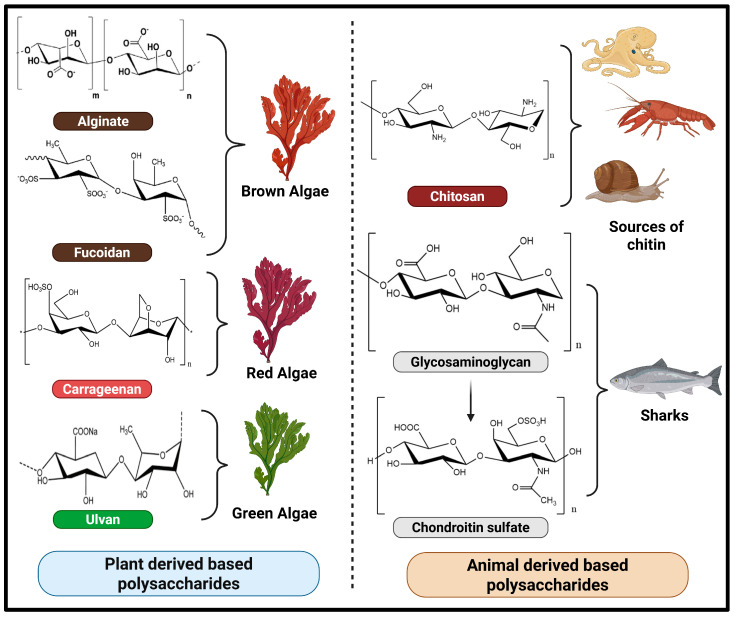
Formulation concept and wound-healing functions of marine-derived polysaccharide-based nanofibers. Marine polysaccharides such as alginate, chitosan, carrageenan, fucoidan, glycosaminoglycans, and ulvan can be fabricated into nanofibrous scaffolds that mimic extracellular matrix architecture, maintain wound moisture, absorb exudate, support gas exchange, and provide localised bioactive signals. Depending on the polymer composition and incorporated therapeutic agents, these nanofibers may contribute to haemostasis, antimicrobial protection, modulation of inflammation, control of oxidative stress, angiogenesis, cell migration, collagen deposition, and extracellular matrix remodelling.

Marine-derived polysaccharide-based nanofibers have emerged as promising biomaterials for wound healing due to their excellent biocompatibility, biodegradability, and inherent bioactivity [68]. Polysaccharides such as chitosan, alginate, carrageenan, and fucoidan can be electrospun into nanofibrous mats that closely mimic the native extracellular matrix, thereby promoting cell adhesion and proliferation (Figure 4). These nanofibers provide a moist wound environment and exhibit hemostatic, antimicrobial, and anti-inflammatory properties. Moreover, their high surface area enables efficient loading and sustained release of growth factors, antibiotics, or phytoconstituents. Marine polysaccharide-based nanofibers provide a multifunctional, sustainable platform for accelerating wound closure and tissue regeneration [69,70]. Table 1 represents marine-derived polysaccharide-based nanofibrous systems for wound healing: composition, fabrication strategy, fibre characteristics, and key biological outcomes.

### 6.1. Alginate

Alginate is a polysaccharide derived from a variety of brown seaweeds, such as *Laminaria hyperborea*, *Laminaria digitata*, *Laminaria japonica*, *Ascophyllum nodosum*, and *Macrocystis pyrifera* [94]. Alginate compounds are widely used in the pharmaceutical industry for applications such as wound dressings and dental impression materials [95]. Alginate has been effectively utilized as a matrix for the entrapment and delivery of biological agents, including drugs and proteins. Proteins can be effectively loaded and released by alginate matrices while maintaining their biological activity, attributed to the mild gelation process of alginate [69]. Alrata et al. reported that alginate dressings develop a gel-like consistency when hydrated, fostering a moist environment suitable for fibroblast proliferation within 2–3 days following a wound injury. Alginate’s biocompatibility is partly attributed to its ability to promote macrophage polarization, which involves a transition from a pro-inflammatory (M1) to a pro-healing (M2) state. The transition of macrophages plays a crucial role in the foreign body response: M2 macrophages facilitate tissue repair and remodeling, whereas M1 macrophages release substances that promote inflammation and contribute to further tissue damage. Alginate seems to create a local wound microenvironment that supports macrophage polarization, functioning not only as an anti-inflammatory agent but also as a promoter of tissue regeneration and stabilization [96]. Wang et al. fabricated nanofiber mats using sodium alginate and performed Western blotting, indicating that these mats may enhance diabetic wound healing by inhibiting the TLR4/NF-κB/NLRP3 signaling pathway, an inflammatory cascade, while upregulating the expression of VEGFA and PDGFA. Inhibition mitigates persistent inflammation, a significant barrier to diabetic wound healing [97]. Ding et al. focused on diabetic wound healing by creating nanofiber mats utilizing sodium alginate. Western blotting experiments showed reduced inflammation through inhibition of the IκBα/NF-κB signalling pathway, resulting in lower levels of pro-inflammatory cytokines. During the wound proliferation phase, VEGFA acts as a pro-angiogenic factor, facilitating wound healing. The expression of HIF-1α and VEGFA was initially low but was significantly upregulated after treatment, thereby promoting angiogenesis during the wound-healing process [98]. Chen et al. developed an alginate/gelatin sponge integrated with curcumin-loaded electrospun fibers to facilitate haemostasis. This study demonstrated that an alginate sponge exhibits strong gelling properties and can exchange sodium ions with blood, forming a sodium alginate gel layer over the wound surface. The exchanged calcium ions subsequently enter the bloodstream, activating clotting components and facilitating rapid haemostasis [99].

### 6.2. Chitosan

Chitosan is obtained by the partial deacetylation of chitin, yielding a linear cationic polysaccharide with free amino groups. These amino groups can be protonated in acidic conditions to form water-soluble chitosan salts, which contribute to its versatile biomedical applications [100,101]. Chitin constitutes the primary component of the exoskeletons of arthropods and crustaceans, including crabs, shrimps, and lobsters, and can also be derived from certain fungi and nematodes. Chitin is insoluble in water; hence, it is typically transformed into soluble derivatives such as chitosan (soluble under acidic conditions) and carboxymethyl chitosan (soluble across a broad spectrum of acidic and alkaline solutions) [102,103]. Li et al. demonstrated that chitosan oligosaccharide (COS)-containing nanofibers enhance wound healing by activating the TGF-β1/Smad (transforming growth factor-beta 1 (TGF-β1)/Small Mothers Against Decapentaplegic) pathway. The results indicated that the PVA/COS-AgNPs nanofiber enhanced wound healing and increased the expression levels of cytokines related to the TGFβ1/Smad signalling pathway, including TGFβ1, TGFβRI, TGFβRII, collagen I, collagen III, pSmad2, and pSmad3. TGFβ is recognized for its significant role in regulating various cellular functions. There are four isoforms of TGFβ. Notably, TGFβ1 is significant in nearly all stages of wound healing and scar formation. Smad family proteins, including Smad2, Smad3, and Smad4, function as mediators of TGFβ1 signalling, facilitating signal transmission from the cytoplasm to the nucleus. The TGFβ1/Smad signaling pathway is intricately linked to wound healing [104]. Liu et al. demonstrated that PVP/CS/DHM (Polyvinylpyrrolidone/Chitosan/Dihydromyricetin) enhances diabetic wound healing by inhibiting the activation of the TLR4/MyD88/NF-κB (Toll-like receptor 4/Myeloid differentiation primary response 88/Nuclear factor kappa-light-chain-enhancer of activated B cells) signaling pathway, resulting in reduced levels of TLR4, MyD88, NF-κB, and the phosphorylated form of IκBα compared to IκBα. This results in a diminished inflammatory response, essential for diabetic wound healing, and enhances the expression of autophagy-related proteins (LC3-II/I, ATG5, ATG7 (Microtubule-associated protein 1 light chain 3, Autophagy-related gene 5, Autophagy-related gene 7)) and markers such as CD31 and HIF-1α (Cluster of differentiation 31 and hypoxia-inducible factor-1 alpha) in skin tissues. This promotes epidermal cell proliferation and migration, re-epithelialization, and accelerated wound closure [105].

### 6.3. Carrageenan

It belongs to a group of high-molecular-weight sulfated polysaccharides extracted from specific red seaweed species. It is mostly sourced from several Rhodophyta species: *Chondrus, Eucheuma, Gigartina, and Hypnea*. It is widely used for its superior physical and functional properties, including gelling, thickening, emulsifying, and stabilizing [106]. Sathuvan et al. identified YAP (Yes-associated protein) and TAZ (Transcriptional activator with PDZ domain) as essential mediators of wound-healing and tissue-regeneration signalling. They serve as significant regulators of cell proliferation and survival, playing essential roles in tissue regeneration. YAP and TAZ function as downstream effectors of the Hippo pathway. YAP and TAZ play essential roles in tissue regeneration by regulating organ growth, stem cell self-renewal, and cell differentiation [107]. Vicens et al. discussed how polysaccharides can modulate cell signaling pathways, cell adhesion, and biological responses. Nonetheless, κ-Carrageenan/PEO films, regardless of the presence of magnesium oxide nanoparticle reinforcement, demonstrated no antibacterial activity against *E. coli* or *P. aeruginosa*, as indicated by the absence of inhibition zones in the Kirby–Bauer assay. The inclusion of 1% MgO-NPs did not provide adequate antibacterial efficacy in the formulated films. Therefore, the activity is contingent on the quantity used in its evaluation [108]. Raghunathan et al. developed microalgal peptide-loaded PCL/κ-carrageenan nanofibers via electrospinning, incorporating bioactive peptides isolated from native microalgae into a polymeric scaffold. The successful fabrication and peptide encapsulation were confirmed by advanced physicochemical characterization, including FTIR, TGA, SEM, and contact angle analysis. The SEM micrographs revealed smooth, uniform, and interconnected bead-like nanofibers, indicating effective fiber formation and homogeneous peptide distribution within the polymeric matrix. Additionally, peptide incorporation (<10 kDa) significantly altered the surface characteristics and reduced the wettability of the nanofibers, which may enhance their interaction with biological tissues. The antimicrobial activity of the fabricated nanofibers was evaluated against *Escherichia coli* (MTTC 443) and *Staphylococcus aureus* (MTTC 96), where a clear zone of inhibition was observed, measuring 24 ± 0.5 mm and 14 ± 0.5 mm, respectively, demonstrating strong antibacterial efficacy. The MIC analysis further supported the concentration-dependent inhibition of bacterial growth. Furthermore, in vitro biocompatibility studies using HEK-293 cell lines confirmed the nanofibers’ cytocompatibility, with enhanced cell viability and proliferation. The wound scratch assay also demonstrated accelerated cell migration and significant wound closure, highlighting the regenerative potential of the developed nanofibrous scaffold. Overall, the study concluded that microalgal peptide-conjugated PCL/κ-carrageenan nanofibers possess excellent antimicrobial, biocompatible, and wound-healing properties, making them a promising biomaterial for wound care and tissue regeneration applications [109]. Gouda et al. developed a novel electrospun nanofibrous scaffold composed of polyvinyl alcohol (PVA), iota-carrageenan (IC), and partially reduced graphene oxide (prGO) for wound healing applications. The scaffold characterisation demonstrated the successful incorporation of prGO, as evidenced by XPS spectra, confirming the presence of carbon and oxygen functional groups, indicating its hydrophilic nature. Raman spectroscopy showed characteristic D and G bands, validating structural defects and graphitic domains of prGO. Further, XRD analysis revealed the transformation into an amorphous scaffold structure, while FTIR spectra confirmed intermolecular hydrogen bonding among PVA, IC, and prGO. Additionally, BET analysis demonstrated a highly porous architecture, and TEM micrographs showed uniform dispersion of prGO within the electrospun nanofibers, enhancing the scaffold’s bioactivity and surface area. In vivo wound-healing analysis further revealed accelerated wound contraction, enhanced re-epithelialization, and improved hair follicle regeneration, confirming the scaffold’s effectiveness as a multifunctional wound dressing [110].

### 6.4. Fucoidan

**Figure 5:**
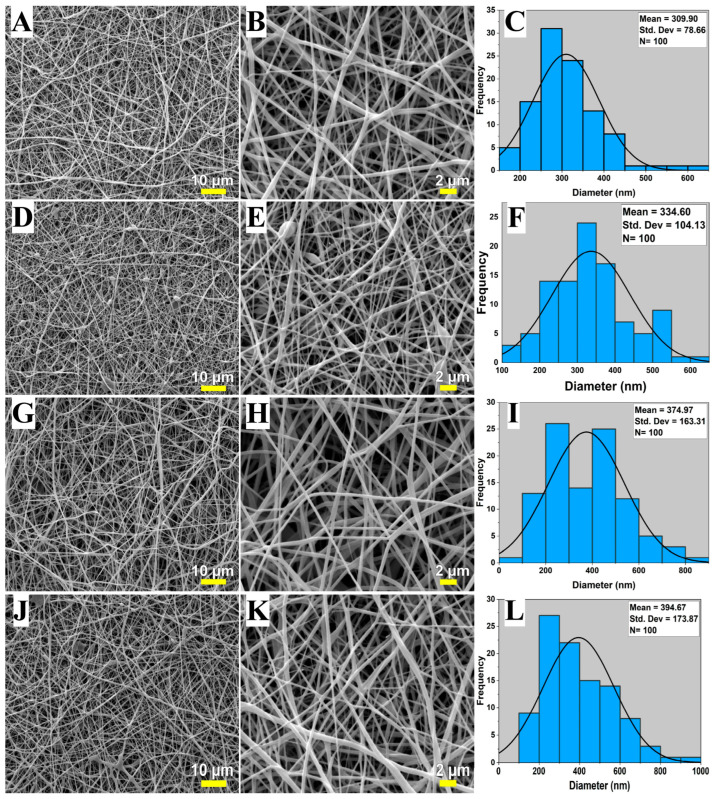
Scanning electron microscopy images and the corresponding distribution of fiber diameters of electrospun nanofibrous mats: PVA-HL (**A**–**C**), PVA-HL-10FUC (**D**–**F**), PVA-HL-13FUC (**G**–**I**), and PVA-HL-15FUC (**J**–**L**) [115].

Fucoidan is a sulfated polysaccharide obtained from the cell walls of brown algae and the tissues of certain marine invertebrates [111]. Fucoidans are derived from the cell walls of various species of brown seaweeds, including *Fucus vesiculosus*, *Cladosiphon okamuranus*, *Sargassum polycystum*, *Laminaria japonica*, and *Undaria pinnatifida*. Fucoidan demonstrates anticoagulant, anti-inflammatory, anti-adhesive, and antiviral properties [112]. Wen et al. investigated the proliferation and tube-formation capabilities of human umbilical vein endothelial cells (HUVECs) subjected to hydrogen peroxide (H_2_O_2_) injury. Their findings indicate that fucoidan enhances wound healing by activating the AKT/Nrf2/HIF-1α signalling pathway (AKT—Protein kinase B (PKB), Nrf2—Nuclear factor erythroid 2–related factor 2, HIF-1α—Hypoxia-inducible factor-1 alpha), thereby promoting angiogenesis. These cells are critical to angiogenesis. The AKT/Nrf2/HIF-1α signalling pathway was evaluated using Western blotting. The pathway was validated using LY294002, a PI3K/AKT inhibitor, which significantly reversed fucoidan-induced angiogenesis, thereby confirming an AKT-dependent mechanism [113]. Phulmogare et al. studied the effects of different concentrations of fucoidan (FD) on the physicochemical characteristics and wound healing of electrospun nanofibers, assessed in vitro and in vivo. As FD content was increased to 1% (upon raising it by 0.25%), the size of nanofibers (diameter) was observed to increase, and the mean diameter went to 627.9 ± 149.78 nm. In addition, the efficiency of drug entrapment was greatly enhanced, reaching up to 94.9 ± 3.1 at higher FD concentrations. Nanofiber water absorption also improved significantly, indicating that the nanofibers have a higher exudate-handling capacity, which is desirable in wound-dressing materials. Remarkably, the enhanced FD incorporation improved swelling behaviour and encapsulation efficiency, while decreasing the in vitro biodegradation rate, indicating enhanced structural stability of the nanofibrous scaffold. The water vapor transmission rate (WVTR) was evaluated to confirm that all formulations had permeability within the optimal therapeutic range, supporting an adequate moisture balance at the wound site. Among the formulations tested, nanofibers with 1% FD in the PVA/DEX matrix exhibited a sustained drug-release profile, indicating that fucoidan remained available longer at the wound interface. Such a controlled-release effect is especially useful for continuous therapeutic action with no frequent dressing changes. An in vivo experiment on rats with full-thickness excisional wounds found that the contraction of the wound was significantly promoted by treatment with 1% FD-loaded PVA/DEX nanofibers (*p* < 0.0001) whilst control groups produced no contraction. The synergistic effect of fucoidin’s bioactivity and the supportive nanofibrous structure is evident in the improved wound closure rate. In general, fucoidan-enriched nanofibers exhibit promising physicochemical properties, sustained-release profiles, and significant wound-healing effectiveness, indicating their potential for future applications in wound dressing [114]. Ismayilova et al. developed fucoidan-loaded electrospun polyvinyl alcohol/*Halomonas* levan (PVA-HL) nanofibrous mats as advanced wound dressing materials. The SEM analysis demonstrated a uniform fibrous network with interconnected porous morphology, while fiber diameter progressively increased with fucoidan loading, indicating successful incorporation of the bioactive compound [Figure 5A–L]. The average fiber diameter increased from 309.9 ± 78.6 nm in the control PVA-HL mat to 394.7 ± 173.8 nm in the highest fucoidan-loaded formulation, as shown in the histogram analysis [Figure 5A,C,F,I,L]. This increase was attributed to enhanced solution viscosity and polymer chain entanglement. The nanofibers exhibited bead-free and continuous structures, which are favorable for mimicking extracellular matrix architecture and promoting cell adhesion. These findings suggest that incorporating fucoidan improved the structural properties of the electrospun scaffold, making it a promising candidate for wound-healing applications [115].

### 6.5. Glycosaminoglycans (GAGs)

Glycosaminoglycans (GAGs), also known as mucopolysaccharides, are large and structurally complex polysaccharides that interact with numerous proteins involved in both normal physiological and pathological processes. They are broadly classified into two groups: sulfated and non-sulfated GAGs. Sulfated GAGs include chondroitin sulfate, dermatan sulfate, heparin/heparan sulfate, and keratan sulfate, whereas hyaluronic acid represents the major non-sulfated form. Certain GAGs, particularly heparin, heparan sulfate, and dermatan sulfate, function as important biological modulators by stabilising growth factors and regulating cell signalling pathways during tissue injury, wound repair, and infection [116]. The marine species and their anatomical components employed for the extraction of various glycosaminoglycans (GAGs) include ascidians (*Styela plicata*, *Phallusia nigra*)—body tissues; heparin-like glycosaminoglycans, dermatan sulfate, bivalve mollusc (*Nodipecten nodosus*)—Connective tissues; Heparan sulfate-like glycosaminoglycan, sea cucumber (*Ludwigothurea grisea*)—Body wall; and Fucosylated chondroitin sulfate [117]. Chondroitin sulfate is utilised in the treatment of osteoarthritis, osteoarthrosis, and occasionally osteoporosis, whereas keratan sulfate is being investigated as a functional component in eye drops for the treatment of corneal dysfunctions [118]. Ghatak et al. studied various signalling pathways modulated by glycosaminoglycans (GAGs), specifically hyaluronan (HA) and GAG chains of proteoglycans, which play a direct role in wound healing and fibrosis. The pathways include HA–CD44 (Hyaluronic acid-Cluster of differentiation 44) signalling, TLR2/TLR4–NF-κB inflammatory (Toll-like receptor 2/Toll-like receptor 4—Nuclear factor kappa-light-chain-enhancer of activated B cells) signalling mediated by HA fragments, modulation of TGF-β (Transforming growth factor-beta) signalling dependent on HA/GAG, PI3K/Akt and MAPK (Phosphoinositide 3-kinase/Protein kinase B (PKB) and Mitogen-activated protein kinase) signalling through CD44 cross-talk, and RHAMM (Receptor for Hyaluronan-Mediated Motility)-mediated signalling [41]. Yang et al. studied various receptor-mediated signalling pathways, including HA–CD44, HA-TLR2/TLR4–MyD88–NF-κB, HA-mediated NF-κB basal survival signalling, HS–Syndecan-4–Integrin–FAK signalling pathway, GAG-mediated MMP regulation pathway, and HS-mediated growth factor signalling (VEGF, FGF, PDGF, and TGF-β). These pathways collectively influence inflammation, angiogenesis, cell migration, and extracellular matrix remodelling, with effects that depend on molecular weight [119]. Cestari et al. developed electrospun nanofibrous dressings composed of silk fibroin (SF), chondroitin sulfate (CS), and silver sulfadiazine (SSD) to provide an effective strategy for controlling wound infections while enhancing patient compliance. To improve the electrospinning process, poly(ethylene oxide) (PEO) was incorporated into the acidic aqueous solution as a temporary spinning aid, facilitating uniform fiber formation. Those pseudo-nanofibers were thoroughly examined with scanning electron microscopy, Fourier transform infrared spectroscopy (FTIR-ATR), energy-dispersive X-ray spectroscopy, differential scanning calorimetry and flame atomic absorption spectroscopy. The mean fibre diameter was 262–328 nm before PEO removal and 242–345 nm after ethanol wash, yielding satisfactory PEO removal without disrupting fibre structure. The elemental and spectroscopic analyses revealed that CS and SSD were retained within the nanofiber matrix. Silver ion quantification showed that CS significantly stabilised SSD, with 75–100% of the drug retained in the fibres. Vero cell-based in vitro cytocompatibility systems reported high cell viability (98–100%), indicating that the cells are non-toxic. Moreover, antibacterial tests disclosed that the greater the CS content, the higher the antimicrobial effect. In vivo testing in Wistar rats showed wound healing with outcomes similar to the standard SSD cream, and the benefit of only one-layer application would reduce the pain of having to change the dressing every two to three days [120].

### 6.6. Ulvan

Ulvan originates from various green seaweed species across multiple nations. The species comprise *Ulva flexuosa*, *Ulva lactuca*, *Ulva meridionalis*, *Ulva ohnoi*, *Ulva rotundata*, and *Ulva armoricana*. The highest ulvan extraction is achieved from two *Ulva* species which are *Ulva armoricana* and *Ulva rotundata*, and it was associated with their active growth period [121]. Kikionis et al. demonstrated that ulvan-based nanofibrous patches enhance wound healing mainly via anti-inflammatory and antioxidant effects, as well as the restoration of skin biophysical parameters; however, the authors did not identify or investigate any specific intracellular wound-healing signaling pathways [122]. Pari et al. explored the NF-κB (Nuclear Factor kappa-B) pathway. Ulvan inhibits IκBα phosphorylation, thereby preventing the nuclear translocation of NF-κB (Nuclear factor of kappa light-chain enhancer of B cells), resulting in decreased expression of pro-inflammatory cytokines such as TNF-α (Tumor necrosis factor alpha), IL-6 (Interleukin-6), and IL-1β (Interleukin-1 beta). This suppression helps regulate excessive inflammation and facilitates tissue repair [123]. Terezaki et al. developed electrospun nanofibrous scaffolds based on ulvan and marine gelatin in different compositional ratios, and thoroughly characterized and investigated their wound healing performance using a second-degree burn model on burn-inflamed skin of SKH-1 hairless mice. To further enhance the therapeutic effect, the potential synergistic effects of incorporating hydrolyzed collagen or silver nanoparticles into ulvan-based nanofibers were also investigated. Clinical, histopathological, and quantitative evaluation of the wound healing potential of the fabricated patches was conducted in a systematic manner based on the evaluation of transepidermal water loss, the level of hydration, epidermal thickness, wound area reduction, the skin texture, and the hemoglobin content. These results indicated that nanofibrous patches made of ulvan and marine gelatin in the most proportionate ratios contributed greatly to wound contraction, especially in the initial stages of healing burn wounds. These formulations were effective in reducing inflammation and promoting homogeneous re-epithelialization, resulting in uniform wound healing. In general, the research indicates that ulvanmarine gelatin electrospun gels are promising effective and bioactive bandages that can be used to treat burn wounds [124].

## 7. Clinical Potential & Translational Challenges

### 7.1. Biocompatibility & Safety Issues

The phases of wound healing are distinct and occur concurrently rather than sequentially; failure in wound healing may arise from a disruption in any of these phases [125]. Contemporary wound care products address deficiencies in the wound-healing process and expedite healing by targeting specific stages. These products create a moist environment conducive to wound healing, prevent and address infection, manage discharge, and alleviate odor and pain associated with the wound.

The existing techniques for loading scaffolding and suture materials with drugs dispersed in polymer carriers facilitate site-specific drug delivery and allow for the modification of release rates. Nonetheless, challenges in regulating polymer degradation rates, loading and distribution of therapeutic agents, ensuring biocompatibility, and acquiring clinical data, among others, hinder further investigation and optimization of these delivery systems [125].

Dressing types include passive (gauze), interactive (hydrogel), advanced (hydrofibers, calcium alginates, hydrocolloids), and antibacterial (iodine and silver dressings). The limitations of passive wound dressings, such as gauze, include the need to soak them in an isotonic saline solution. However, upon evaporation of water, the solution becomes hypertonic, causing fluid to be drawn into the gauze. Without re-moistening the gauze, the packing rapidly dries, forming a hard mass that can make removal painful for the patient [126]. The limitations of interactive wound dressings, such as hydrogels, include poor mechanical strength, difficulty controlling the degradation rate, limited cell adhesion, and the need for secondary dressings to ensure adherence [127,128]. The most commonly used antibacterial wound dressings include silver and iodine dressings, each with specific limitations. Notably, exposure to silver nanoparticles (AgNPs) has been associated with a dose-dependent increase in early apoptosis, indicating that prolonged or high-dose exposure may harm adjacent healthy tissue. Furthermore, the antimicrobial efficacy and cytotoxicity of these dressings are closely linked, which complicates their design. Iodine dressings, in particular, exhibit cytotoxic effects on cells such as keratinocytes and leukocytes, which can impede wound healing. Therefore, it is suitable solely for short-term use [127,129].

There is a need for improved wound-healing materials that can combat infection and enhance tissue repair without antibiotics, to which bacteria are increasingly developing resistance. To address this issue, numerous researchers are conducting studies using electrospinning to create biologically inspired, two-layered nanofibrous polymeric wound dressing mats. This design facilitates dual functionality, with each layer serving a distinct purpose when applied to a wound. The upper layer of the mat exposed to the environment may incorporate antibacterial nanoparticles to inhibit the entry of environmental microorganisms into the injured site. The underside of the mat in contact with the wound can be infused with agents that promote tissue regeneration. A middle layer may be incorporated to mitigate chronic inflammation. Composite materials of this nature can enhance the effectiveness of wound-healing dressings, a priority for future research [127].

### 7.2. Scale-Up Challenges in Electrospinning

The rapid advancement in nanotechnology has resulted in various techniques for producing numerous nano-scale composites, with nanofibers gaining significant attention due to their diverse fabrication technologies and applications, particularly in pharmaceutical drug delivery and biomedical fields such as wound dressing and tissue engineering [130].

Electrospinning techniques are categorised into two configurations: needle electrospinning and needleless electrospinning. Needle electrospinning is the most extensively studied configuration. This configuration has several disadvantages, including needle clogging, low production capacity, and time-consuming operations, all of which reduce production efficiency at both pilot and industrial scales. Conversely, in the needleless configuration, the needle is substituted with a rotating drum that directly contacts the dope solution bath. Most of the identified disadvantages can be mitigated by using needleless electrospinning. The needleless electrospinning technique has garnered increasing attention for forming nanofibers from a diverse array of polymers, in contrast to conventional electrospinning [131,132].

Electrospinning is an economical method for producing dried fibres by employing electrostatic forces on a liquid feed to create ultrafine fiber structures, typically less than 10 μm, which can dry instantaneously at room temperature during operation [133]. The fundamental laboratory apparatus for electrospinning comprises three primary components: (i) a high-voltage power supply, (ii) a spinneret (a metallic needle), and (iii) a metallic collector. Wherein the fibre-forming excipient (such as a polymer, cyclodextrin, or lipid) is dissolved in a solvent, and the resulting solution is delivered into a single spinneret at a consistent, regulated flow rate. A high voltage is applied between the spinneret and the grounded collector, resulting in the formation of a Taylor cone. When electrostatic forces surpass surface tension, a liquid jet emerges from the cone and stabilises as a continuous stream between the nozzle and the collector. During the process, the jets elongate, acquiring a fibre-like structure, while the solvent evaporates rapidly due to the high surface area, as the fibres are typically submicron-sized [133].

Electrospinning is well established at laboratory and pilot scales; however, the authors highlight that scaling up to industrial-level production presents significant challenges, especially in pharmaceutical applications where consistency and volume are crucial. The frequently cited scale-up challenges in the literature include low throughput and productivity, needle clogging and maintenance issues, and solvent handling and safety concerns. Industrial-scale electrospinning necessitates sophisticated, commercially available systems, such as multi-nozzle or needleless configurations. The requirement for specialized equipment elevates both capital expenditures and operational requirements [134,135].

The literature discusses blend electrospinning for protein delivery, noting that many synthetic polymers (e.g., PLGA, PCL, polyurethane) require organic solvents, which can adversely affect protein bioactivity by altering their conformation. The report notes that severe processing conditions, such as solvent exposure, pose significant challenges for the encapsulation of proteins, peptides, and other sensitive biomolecules, as these conditions can denature and inactivate them [136]. Electrospinning frequently relies on volatile solvents such as ethanol, dichloromethane, and methanol. The presence of substantial free liquid surfaces in numerous needleless systems leads to significant solvent evaporation, thereby heightening safety, environmental, and process-control issues for industrial applications [137].

### 7.3. Regulatory Considerations

The lack of specific regulatory guidelines for nanomedicines and health-related nanomaterials creates challenges in risk assessment and safety evaluation. This gap also complicates regulatory decision-making and may delay product development and approval [138]. A significant regulatory challenge involves the precise classification of electrospun products as either medical devices or medicinal products (drugs/biologics). Incorrect classification can invalidate the entire development and approval process [139]. The FDA categorises devices into Classes I, II, and III, reflecting their associated risk levels and regulatory oversight requirements. Class II medical devices, which pose a moderate risk, must comply with both general and specific regulations. Class III devices are essential for safeguarding human health and are typically utilized to sustain human life. These products are classified as the highest risk and require FDA premarket approval (PMA) prior to marketing. The PMA is a scientific and regulatory assessment procedure commonly used to evaluate the safety and efficacy of Class III medical devices. The document is divided into two sections: one addressing clinical investigations and the other focusing on nonclinical laboratory studies. Clinical investigations must comply with the ethical standards, data integrity, and quality criteria established by Good Clinical Practice (GCP). Part 814 of Title 21 in the Code of Federal Regulations (CFR) outlines the regulations for premarket approval [140].

Electrospun scaffolds integrated with drugs, growth factors, peptides, and biomolecules derived from humans or animals establish complex regulatory pathways. If a medicinal substance exhibits ancillary action, the product is classified as a Class III medical device (highest risk). If the primary action is medicinal, the product is classified as a medicinal product. These devices necessitate prior evaluation of their technical data by a recognised regulatory organization [139].

### 7.4. Cost, Sustainability & Marine Resource Utilization

Advanced extraction techniques such as Microwave-Assisted Extraction (MAE), Ultrasound-Assisted Extraction (UAE), Supercritical Fluid Extraction (SFE), Pressurised Solvent Extraction (PSE/PLE), Pulsed Electric Field-Assisted Extraction (PEF), and Enzyme-Assisted Extraction (EAE) notably reduce extraction time, solvent consumption, and energy consumption; however, their high equipment and operational costs restrict industrial adoption. Conventional techniques such as maceration, Soxhlet extraction, hydrodistillation, and decoction/infusion remain widely used because they require minimal capital investment. Enzyme-assisted and ultrasound-assisted extractions are considered more economically viable alternatives for the large-scale extraction of marine compounds [141].

Next-generation materials are being developed by investigating renewable nanofibers, driven by the increasing demand for sustainable alternatives. These nanofibers exhibit several advantageous properties, including enhanced biodegradability, reduced environmental impact, and the potential for resource regeneration. Renewable nanofibers are essential for addressing the growing demand for environmentally friendly solutions, with applications including tissue engineering, environmental remediation, energy storage, and sustainable packaging [142].

Green electrospun nanofiber materials are gaining significance in biomedical and biotechnological applications due to their inherent advantages over conventionally produced nanofibers. Green techniques facilitate the production of nanofibers utilising nontoxic, renewable biomaterials, including collagen, gelatin, chitosan, and cellulose. It involves the utilization of natural polymers (Chitosan, alginate, etc.), biodegradable synthetic polymers (polycaprolactone (PCL), polyhydroxyalkanoates (PHAs), polylactic acid (PLA)), and hybrid and composite nanofibers [142,143].

The marine ecosystem, characterized by high biodiversity, serves as a significant source of bioactive compounds with varied structures and biological properties [144]. Marine polysaccharides are primarily used in the food and cosmetic industries; however, they are also prevalent in the pharmaceutical sciences, with growing interest in their use as materials for incorporating bioactive agents [145]. Marine polysaccharides exhibit a wide variety of structures and remain underutilised, making them a significant source of chemical diversity for drug discovery. Marine algae (brown, red, and green) are the main sources of bioactive polysaccharides, including alginate, carrageenan, fucoidan, and ulvan, whereas marine animals include chitosan, hyaluronan, and chondroitin sulfate. Marine microorganisms represent a novel and controllable source of polysaccharides. These materials demonstrate high biocompatibility, biodegradability, antimicrobial, anti-inflammatory, and regenerative properties, making them well-suited for drug delivery, wound healing, and tissue engineering applications [146].

### 7.5. Sterilisation, Storage, and Raw Material Standardisation Challenges

Despite the promising therapeutic potential of marine-derived polysaccharide-based nanofibrous wound dressings, several practical challenges continue to hinder their large-scale clinical translation [147]. Among these, sterilisation compatibility, storage stability, and raw material standardisation remain critical yet often overlooked barriers. These factors directly affect the physicochemical integrity, biological activity, reproducibility, and regulatory acceptability of marine polysaccharide-based biomaterials [148]. Sterilisation is an essential prerequisite for biomedical wound dressings; however, conventional sterilisation methods may significantly alter the structural and functional properties of natural polysaccharides [149]. Techniques such as autoclaving, gamma irradiation, ethylene oxide treatment, and ultraviolet irradiation are commonly employed, but each presents specific limitations. Autoclaving involves high temperature and pressure, which may induce hydrolysis, depolymerization, and viscosity loss in heat-sensitive polymers such as alginate and chitosan [150]. Gamma irradiation, although highly effective and widely accepted for sterilization, can cause chain scission, molecular weight reduction, and alteration of sulfation patterns in fucoidan and carrageenan, ultimately affecting their biological activity and mechanical stability. Ethylene oxide sterilization is suitable for thermolabile biomaterials but may leave toxic residues that require extensive aeration before biomedical application [151]. Ultraviolet sterilization offers only surface-level sterilization and is inadequate for thick or multilayer nanofibrous scaffolds. Therefore, selecting an appropriate sterilization strategy is highly dependent on polymer chemistry, scaffold architecture, and the sensitivity of incorporated bioactive compounds.

Storage stability is another major translational concern, particularly because marine polysaccharides are highly hygroscopic and structurally dynamic [152]. Exposure to environmental moisture, oxygen, temperature fluctuations, and light may trigger hydrolysis, oxidation, and conformational changes that compromise scaffold integrity. Chitosan-based systems are especially sensitive to humidity, which may alter their swelling behavior and mechanical strength over time [153]. Alginate may undergo ionic exchange and loss of gel-forming capacity during prolonged storage [154], whereas sulfated polysaccharides such as fucoidan and carrageenan may experience partial desulfation, reducing their antioxidant, anti-inflammatory, and angiogenic potential [155]. Additionally, nanofibrous dressings loaded with therapeutic agents may exhibit premature drug leakage or altered release profiles during storage. These stability concerns necessitate controlled storage conditions such as low humidity, inert atmosphere packaging, and temperature-regulated environments to preserve material performance.

One of the most significant challenges associated with natural marine polysaccharides is the lack of standardisation of raw materials. Unlike synthetic polymers, marine polysaccharides exhibit considerable batch-to-batch variability depending on species, geographical origin, seasonal variation, extraction method, and purification process [156]. For instance, chitosan derived from different crustacean sources may vary substantially in molecular weight, degree of deacetylation, crystallinity, and impurity profile, all of which influence solubility, electrospinnability, antimicrobial activity, and biodegradation behavior [157,158]. Similarly, alginate composition varies according to the mannuronic acid to guluronic acid (M/G) ratio, which determines gelation properties, mechanical strength, and cell interactions [159]. Fucoidan and carrageenan also exhibit variability in degree of sulfation, monosaccharide composition, and branching patterns, directly influencing their biological functions [155]. Such heterogeneity creates significant challenges in ensuring reproducibility, biological consistency, and therapeutic predictability.

## 8. Future Directions

### 8.1. Bioinspired & Multifunctional Nanofibers

For millennia, the oceans have been valued for their supply of efficient transportation and abundant food resources. Consequently, it is evident that modern society will have to continue using the oceans and maximise their benefits. There may be significant treasures of valuable materials and novel bio-compounds. The synthesis of established and emerging marine sectors is referred to as the Blue Economy [160]. The seas constitute the planet’s largest ecosystem, covering about 70% of Earth’s surface. Marine ecosystems harbour an extensive diversity of species, many of which remain unexplored. According to the Census of Marine Life, at least 50% and potentially more than 90% of marine species are yet to be scientifically described. Marine sources, including microbes, symbionts, extremophiles, fungi, plants, and animals, are scientifically significant due to their role in the creation of marine biomolecules, namely biocatalysts, which encompass all marine bioprocesses [161]. Researchers have developed materials with numerous applications in human health, agriculture, energy, aquaculture, fine chemicals, pharmaceuticals, food, cosmetics, and environmental fields, including biosensing and bioremediation. Biomimetic techniques are increasingly attracting attention, and the scope of research focused on marine-derived or marine-inspired materials is consistently expanding [162].

Marine compounds derived from animal, algal, or microbial sources exhibit a wide range of properties and are used across several application domains. Marine natural products, ranging from small bioactive molecules (mostly secondary metabolites) to big biomacromolecules (proteins and polysaccharides), have seen a growing application in cosmetics, as well as in medicine for the treatment of cancer and chronic diseases, and in cell therapy and tissue engineering [146,160,162,163]. Furthermore, marine polymers represent particularly viable substitutes for synthetic polymers in the creation of sustainable and environmentally friendly materials, such as bioplastics and biocomposites [163]. Glycosaminoglycans (GAGs) and chitin, both originating from animals, are extensively researched and being utilized in multiple fields, some emphasis will be on hyaluronic acid (HA—a type of glycosaminoglycan) [162]. Hyaluronic acid (HA) is a glycosaminoglycan present in extracellular tissue throughout different areas of the body. This material is gaining significance in biomaterials science and is being utilized in several applications, including tissue culture scaffolds and cosmetic products. Hyaluronic acid (HA) scaffolds offer numerous advantages, including natural biocompatibility and in vivo enzymatic degradation without producing toxic by-products, making them safe for tissue engineering and wound-healing applications. HA plays a crucial role in all phases of the wound healing process. Furthermore, HA can be fabricated into hydrogels, sponges, meshes, and porous scaffolds through various techniques such as freeze-drying, electrospinning, bioprinting, and phase separation, thereby enhancing its applicability across different tissues [162,164]. Hyaluronic acid can be collected from marine organisms, including stingray livers, bivalves, and fish tissues such as cartilage matrix and vitreous humor, the latter being considered as the most suitable source [165,166]. Chitin was first extracted from mushrooms by the French botanist H. Braconnot in 1811. It is among the most prevalent polysaccharides in nature [162]. This structural biopolymer is present in several organisms (animals, algae, and fungi) as structured crystalline microfibrils. It is the primary component of the exoskeleton in insects, mollusks, and crustaceans [167]. Chitin is presently extracted on an industrial or semi-industrial scale from the shells of shrimp, crabs, and lobsters, which are readily accessible and plentiful by-products of the shellfish processing industry. It is biodegradable; however, it suppresses certain microbial activities, and is biocompatible, exhibiting low cytotoxicity and immunogenicity [168].

### 8.2. Personalized Wound Dressings

Wound dressings are very significant for severe skin damage. Using burns as an example, wound dressings remain essential in the therapy process. Wound dressings effectively protect the wound bed from external factors during initial first-aid treatment after trauma [169]. Non-healing wounds such as pressure ulcers, leg ulcers, diabetic foot ulcers, and chronic surgical wounds commonly arise due to underlying systemic and local factors. These include advanced age, prolonged immobility or paralysis, diabetes mellitus, peripheral arterial or venous insufficiency, vasculitis, sickle cell anemia, immunosuppression, renal dysfunction, autoimmune conditions, and various dermatological disorders. Collectively, these comorbidities impair normal wound-healing mechanisms, leading to delayed or incomplete tissue repair [170]. Wound healing is complicated or delayed by two categories of factors: systemic and local. Systemic considerations encompass age, body composition, chronic illnesses, immunosuppression, dietary condition, radiation treatment, and vascular deficiencies whereas local variables and their etiologies encompass desiccation, infection, aberrant bacterial proliferation, maceration, necrosis, pressure, trauma, and edema [171,172]. At present, clinicians subjectively evaluate local factors by visual inspection and, less frequently, by laboratory analysis of exudate swabs. The persistent increase in expenditures and the growing prevalence of chronic wounds illustrate that the old and reactive methodology in wound care is ineffective, necessitating the adoption of a proactive prevention strategy. This requirement can be met by new technologies that provide clinicians with non-invasive, quantitative wound data, enabling continuous wound monitoring and enhancing both wound assessment and characterization. These ‘smart’ dressings will merge multi-parametric detection of wound characteristics. This significant advancement in wound care will primarily cut healing times and enable remote patient monitoring. The results will include quicker healing, diminished complications, short inpatient durations, decreased attendance at public health clinics and General Practitioners, and a reduction in necessary home visits, culminating in overall cost reductions for wound care delivery. This will reduce superfluous expenditures in this domain, ultimately freeing up essential healthcare funds [173,174]. The authors highlight that conventional “one-size-fits-all” medication manufacturing fails to account for individual patient variability, whereas 3D printing enables the production of on-demand, patient-specific pharmacological formulations with precise control over dosage, shape, and release characteristics. Three-dimensional printing enables the digital design and modification of drugs using software, allowing rapid adjustments to dosage strength, shape, size, internal structure, and release profile to meet specific patient requirements. This feature immediately facilitates personalized medicine by enabling variable and decentralized medication synthesis, rather than mass-produced standard formulas [175]. The advantages of personalized medicine can be categorized into three primary domains: (i) delivering better therapies to patients, (ii) yielding benefits for healthcare systems and society, and (iii) facilitating more efficient development of new drugs. This has led to an increased probability of therapeutic efficacy, improved outcomes, and a diminished risk of adverse events for patients. The advantages for the healthcare system and society are clear, as evidenced by improvements in patient care and cost reduction through reduced reliance on inefficient treatments, lower expenses associated with chronic illnesses, and shorter hospitalizations [176].

Teoh et al. conducted a study on personalized wound dressings using 3D extrusion printing to customize the shape, size, medication type, drug dosage, and drug release profile based on individual patient and wound specifications. The dressing was initially designed using CAD software to precisely match the size and shape of the patient’s wound, then fabricated via 3D printing. The drug dose is administered by adjusting the dimensions of the wound dressing and the number of printed layers. The 3D printer uses multiple printheads, enabling simultaneous administration of various pharmaceuticals [177]. Muwaffak et al. successfully produced patient-specific wound dressings by incorporating zinc, copper, and silver into an FDA-approved polymer, polycaprolactone (PCL). Research indicates that integrating 3D scanning with 3D printing can produce customized wound dressings that conform to anatomically intricate body regions (e.g., the nose and ear), offering a superior alternative to traditional surface dressings. A rapid release in the first 24 h, followed by a gradual, continuous release over 72 h, was effectively achieved [178].

### 8.3. Next-Generation Marine Biomaterials

The current pharmaceutical research landscape, marked by a growing array of small-molecule and biologic drug candidates, necessitates advanced delivery technologies to address issues of bioavailability, stability, and patient compliance. One of the most promising areas in this research is the valorization of marine biomass, particularly seafood processing by-products, as a source of bioactive polysaccharides and proteins for medication delivery and wound-healing applications [179]. Khrunyk et al. discussed marine polysaccharides (chitin, alginates, fucoidans, carrageenans, ulvans, agar) and marine structural proteins (spongin, collagen, gelatin, keratin) as sophisticated, multifunctional marine biomaterials, stating their potential as next-generation materials owing to their biomimetic architectures, bioactivity, sustainability, and relevance in wound healing and regenerative medicine [180]. Crustaceans (lobster, crab, and krill), combined with commercial chitin, can serve as biosorbents for removing heavy metals from surface runoff, addressing two environmental issues: the utilization of seafood waste and the management of water resources [181]. Heras et al. reported the development of chitin-infused sponge-like scaffolds that were biocompatible with human mesenchymal stromal cells (hMSCs), demonstrating significant promise for biomedical applications, particularly in tissue engineering [182]. Ultimately, nanomaterials derived from shrimp chitin (nanocrystals and nanofibers) were found to be non-cytotoxic, as assessed using epithelial- and fibroblast-like cell lines [183]. In addition, study of chitinous scaffolds derived from *Lanthella* species revealed their elasticity and capillary effect, enabling these distinctive matrices to conform to the shapes of objects they contact and absorb liquids, such as blood, properties that can be utilized in wound treatment. Historically, the worth of marine macroalgae (seaweeds) was significantly undervalued. In ancient Greece, Virgil and Horace employed the word “vilior alga” to denote something entirely useless [180].

Seaweeds can be categorized as green, red, and brown algae, and they contain various polysaccharides whose properties have been thoroughly investigated over the past decade. Nearly all brown algal species are marine, predominantly found in cold waters, particularly at northern latitudes, and are abundant in polysaccharides such as alginates [180,184,185]. Alginates are widely used as polymeric coatings and carriers for therapeutic compounds, functioning as hydrogels or composites, and are widely applied in tissue engineering [180]. Additionally, alginate-based wound dressings are distinguished by their ability to maintain a moist environment and mitigate bacterial infections, both of which are critical for effective wound healing [186]. Marine-derived biological materials constitute a distinct scientific niche within worldwide biomaterials research, characterized by a lengthy history of investigation and use across various human endeavours. Processed marine biological waste is increasingly regarded as a raw resource for biomaterial synthesis, which, unlike synthetic counterparts, exhibits superior biocompatibility and great biodegradability. Advances in marine biomaterials research are primarily driven by its robust interdisciplinary nature: collaboration among experts in marine and structural biology, bioinspired materials chemistry, biomineralogy, biomimetics, biomechanics, and solid-state physics is essential to enhance the scientific and practical dimensions of this field.

## 9. Structure–Activity Relationship of Marine-Derived Polysaccharide Nanofibers in Wound Healing

The therapeutic performance of marine-derived polysaccharide nanofibers is determined not solely by their intrinsic bioactivity but by a complex interplay between their molecular architecture, physicochemical characteristics, and nanofibrous scaffold morphology. This structure–activity relationship (SAR) governs critical biological processes, including hemostasis, immunomodulation, angiogenesis, extracellular matrix (ECM) remodeling, antimicrobial activity, and tissue regeneration. Therefore, understanding how specific structural attributes influence biological responses is essential for the rational design of next-generation wound dressings with predictable therapeutic efficacy.

Among marine polysaccharides, chitosan represents one of the most extensively investigated polymers because its biological functions are highly dependent on its degree of deacetylation (DDA) and molecular weight [187,188]. Increasing the DDA exposes a greater number of protonated amino groups, producing a highly cationic polymer capable of electrostatically interacting with negatively charged bacterial cell membranes [189,190]. This interaction disrupts membrane integrity, resulting in broad-spectrum antimicrobial activity while simultaneously promoting platelet adhesion, accelerating hemostasis, and enhancing fibroblast attachment. Furthermore, positively charged chitosan surfaces facilitate macrophage polarization toward the regenerative M2 phenotype, thereby suppressing excessive inflammatory signaling and promoting tissue remodeling [191,192]. Conversely, lower-molecular-weight chitosan generally exhibits greater aqueous solubility and faster biodegradation, enabling more efficient release of encapsulated therapeutic agents and facilitating rapid cellular infiltration during the early stages of wound repair.

In contrast, the biological performance of alginate is largely influenced by the relative abundance of β-D-mannuronic acid (M) and α-L-guluronic acid (G) residues [96,193]. Alginates enriched in guluronic acid exhibit greater affinity for divalent calcium ions, forming mechanically robust “egg-box” structures that enhance nanofibrous stability during prolonged wound treatment [194]. Such nanofibrous maintain structural integrity under hydrated conditions and provide sustained support for granulation tissue formation [195]. Conversely, alginates with higher mannuronic acid fractions exhibit increased flexibility, swelling capacity, and water retention, thereby enhancing exudate absorption and maintaining an optimal moist microenvironment for cell migration and re-epithelialization [196]. Consequently, the M/G ratio directly influences degradation behavior, mechanical performance, and biological functionality.

For sulfated marine polysaccharides such as fucoidan and carrageenan, the density and spatial distribution of sulfate groups represent critical structural determinants of biological activity [155,197]. Sulfate residues impart a strong negative surface charge that facilitates electrostatic interactions with numerous heparin-binding growth factors, including vascular endothelial growth factor (VEGF), fibroblast growth factor (FGF), platelet-derived growth factor (PDGF), and transforming growth factor-β (TGF-β) [198,199]. These interactions protect growth factors from proteolytic degradation while prolonging their localized bioavailability within the wound bed, thereby promoting angiogenesis, fibroblast proliferation, collagen synthesis, and extracellular matrix remodeling. In addition, increased sulfation enhances antioxidant capacity by suppressing reactive oxygen species (ROS)-mediated damage and attenuating NF-κB-dependent inflammatory signaling while simultaneously activating cytoprotective pathways such as Nrf2/HO-1 [200]. Therefore, sulfate content is an important molecular parameter that regulates both anti-inflammatory and pro-regenerative activities.

## 10. Conclusions

Marine-derived polysaccharide-based nanofibers represent a biologically attractive and technologically flexible platform for advanced wound care. Their relevance is not limited to structural mimicry of the extracellular matrix; rather, their value lies in the possibility of combining scaffold architecture with intrinsic or loaded bioactivities that support haemostasis, microbial control, inflammation resolution, redox balance, angiogenesis, fibroblast activity, keratinocyte migration, collagen deposition, and extracellular matrix remodelling. Among the major marine polysaccharides, alginate, chitosan, carrageenan, fucoidan, glycosaminoglycans, and ulvan offer different but complementary functional profiles, making them suitable candidates for multifunctional wound dressings, particularly in chronic, infected, diabetic, burn, and exudative wound environments.

Despite this promise, the current evidence base remains strongly weighted toward in vitro assays and small preclinical studies. Translation is still limited by variability in marine-polymer sources and extraction and purification methods; molecular weight; sulfation or deacetylation degree; batch-to-batch reproducibility; mechanical weakness; sterilisation effects; storage stability; scalable electrospinning; and incomplete toxicological and regulatory evaluation. Therefore, future work should move beyond proof-of-concept scaffold fabrication and prioritise standardised material characterisation, comparative testing against clinically used dressings, infected and diabetic wound models, long-term safety assessments, reproducible manufacturing protocols, and early clinical validation. In this context, marine-derived polysaccharide nanofibers should be viewed as promising translational candidates rather than clinically established solutions. Their impact on wound care will depend on whether future studies can convert their strong mechanistic rationale into reproducible, safe, scalable, and clinically meaningful therapeutic performance.

## Figures and Tables

**Table 1:** Marine-derived polysaccharide-based nanofibrous systems for wound healing: composition, fabrication strategy, fiber characteristics, and key biological outcomes.

Polymers	Drug Used	Technique Used	Dimension	Special Outcome	References
Sodium alginate	Betamethasone	Solution electrospinning (single-needle electrospinning)	Average fibre diameter ranged between ~165–187 nm	The electrospun sodium alginate/gelatin nanofibrous films loaded with betamethasone exhibited a multifunctional wound-healing profile, combining anti-inflammatory activity, biocompatibility, and hemostatic potential. Their nanoscale fibrous architecture and favourable physicochemical properties suggest strong potential as an advanced bioactive wound dressing for accelerated skin regeneration.	[71]
Sodium alginate/PLGA	Ciprofloxacin	Solution electrospinning of a suspension system (single-needle electrospinning	Fibers ranged between ~747–877 nm	The ciprofloxacin-loaded PLGA/alginate electrospun mats exhibited enhanced multifunctionality by integrating moisture retention, improved drug release, and antimicrobial efficacy, addressing key limitations of conventional wound dressings. The synergistic combination of hydrophobic PLGA and hydrophilic alginate offers a promising strategy for developing advanced wound care systems with optimised mechanical and therapeutic properties.	[72]
Sodium alginate/PVA	Ciprofloxacin	Solution electrospinning (single-needle electrospinning)	CIP-loaded sodium alginate/PVA fibers ranged between 200–300 nm	The ciprofloxacin-loaded PVA/NaAlg electrospun nanofibrous patch showed significant potential for rapid and localised wound management by ensuring sustained antibiotic release and enhanced healing efficacy. Increased hydroxyproline content and faster wound contraction in vivo confirmed its superior regenerative performance, making it a promising biomaterial for acute wound care applications.	[73]
Sodium alginate (SA) + Polycaprolactone (PCL)	Methylene blue (MB) and methyl orange (MO) were used as model drug molecules	Solution electrospinning	The PCL layer was 300 ± 50 nm and the Alginate layer 100 ± 30 nm.	The multilayer alginate–polycaprolactone nanofibrous membrane embedded with ZnO nanoparticles demonstrated excellent mechanical stability, exudate management, and tunable drug release properties, making it highly suitable for advanced wound care. Its dual-layer architecture combines protective barrier functions with enhanced tissue regeneration, offering a cost-effective and scalable platform for multifunctional wound healing applications.	[74]
Sodium alginate \/Polyvinyl alcohol (PVA)/Poly(lactic-co-glycolic acid) (PLGA)	*Capparis sepiaria* aqueous root extract	Solution electrospinning (single-needle electrospinning)	Nanofibers exhibited diameters in the range of ~1.15–1.85 µm, depending on formulation.	The *Capparis sepiaria*-loaded SA/PVA and SA/PVA/PLGA electrospun nanofibers demonstrated potent antibacterial, hemostatic, and wound-healing activities, highlighting their effectiveness against burn-wound-associated complications such as infection and excessive exudation. Their ECM-mimicking porous architecture, combined with the bioactive phytochemicals in the plant extract, makes them a promising natural, multifunctional platform for accelerated burn wound regeneration.	[75]
Sodium alginate (SA)/Poly(vinyl alcohol) (PVA)	Gatifloxacin hydrochloride	Solution electrospinning (single-needle electrospinning)	-----	The gatifloxacin-loaded sodium alginate/PVA electrospun nanofibers demonstrated efficient drug encapsulation with an initial rapid release followed by sustained delivery, making them suitable for controlled antimicrobial therapy in wound healing. Their uniform fibrous morphology and strong polymeric interactions highlight their potential as a biocompatible and effective drug-delivery platform for infection management and accelerated tissue repair.	[76]
Sodium alginate (AL) + Poly(ethylene oxide) (PEO)	Ciprofloxacin hydrochloride	Solution electrospinning	Average diameter of the fibers ranged from 109 nm (unloaded fibers) to 161 nm (loaded fibers)	The ciprofloxacin-loaded alginate nanofibers exhibited uniform nanoscale morphology and controlled antibiotic release, demonstrating their potential as effective wound dressings for localised infection management. The successful removal of PEO after cross-linking and the predominance of Fickian diffusion highlight the structural stability and sustained drug-delivery capability of the alginate-based nanofibrous system.	[77]
Sodium alginate (SA) + Chitosan	Silver nanoparticles act as the antibacterial agent	Solution electrospinning	-----	The chitosan-mediated silver nanoparticle-coated alginate electrospun membrane demonstrated strong antibacterial activity against both Gram-positive and Gram-negative bacteria, making it highly effective for infection prevention in wound management. Its stable polyelectrolyte complex structure, combined with suitable water vapor transmission and biocompatible properties, highlights its potential as an advanced antimicrobial wound dressing.	[78]
Poly(vinyl alcohol) (PVA)/Sodium alginate (Alg)/Poly(acrylic acid) (PAA).	Ciprofloxacin	Solution electrospinning (single-needle electrospinning)	Diameter distribution was 141 ± 53 nm	The PVA/alginate/PAA nanofibrous matrix loaded with ciprofloxacin demonstrated superior mechanical strength, sustained drug release, and prolonged antibacterial activity, making it highly effective for infection-controlled wound healing. Its enhanced re-epithelialization and faster tissue regeneration in vivo highlight the multifunctional polymeric network’s synergistic potential as an advanced wound dressing system.	[79]
Zein (corn protein)/Fucoidan	Fucoidan	Electrospinning	234–276 nm	The zein–fucoidan electrospun ultrafine fibres demonstrated excellent biocompatibility, favourable hydrophilicity, and optimised nanoscale morphology, making them promising candidates for wound healing and skin adhesive applications. The incorporation of fucoidan improved fiber architecture and may enhance biological performance, highlighting the potential of combining renewable proteins with marine polysaccharides for advanced biomedical materials.	[80]
Polyvinylpyrrolidone (PVP) with κ-carrageenan blend	Ursolic acid (UA)	Electrospinning	Nanofibers exhibited average fibre diameters of ~97–99 nm.	The ursolic acid-loaded PVP/κ-carrageenan electrospun nanofibers demonstrated superior wound-healing performance, with enhanced porosity, hydrophilicity, antioxidant activity, and antibacterial efficacy. Their ability to significantly enhance cell proliferation and accelerate wound closure highlights their strong potential as multifunctional bioactive wound-dressing materials.	[81]
Gelatin/Carrageenan	Platelet-rich fibrin (PRF) (source of growth factors PDGF-AB and VEGF)	Freeze-drying (lyophilisation) for gelatin sponge/Electrospinning for carrageenan	Exhibited diameters in the ~130–245 nm range	The gelatin–carrageenan bilayer dressing incorporated with platelet-rich fibrin demonstrated enhanced mechanical strength, sustained growth factor release, and superior angiogenic potential, closely mimicking the hierarchical structure of native skin. Its remarkable wound closure rate and improved histopathological regeneration highlight its strong potential as an advanced bioactive dressing for full-thickness wound healing.	[82]
Polycaprolactone (PCL)/Ulvan (ULV)	No-drug	Electrospinning	296–411 nm depending on ulvan content	The polycaprolactone–ulvan electrospun composite mat demonstrated improved hydrophilicity, enhanced fibroblast adhesion and proliferation, and favourable regulation of wound remodelling genes, indicating its strong potential for scarless wound healing. The incorporation of ulvan into the PCL matrix successfully mimicked extracellular matrix properties, making it a promising bioactive scaffold for advanced wound dressing applications.	[83]
Poly(ε-caprolactone) (PCL)/Chitosan (CS)	Curcumin	Electrospinning combined with electrospraying	32.17 ± 0.39 nm	The curcumin nano-encapsulated PCL/chitosan electrospun nanofiber demonstrated enhanced antibacterial, antioxidant, and cell proliferation activities, significantly accelerating wound healing in MRSA-infected wounds. Its improved swelling behavior, water vapor transmission, and organized tissue regeneration highlight its potential as an effective multifunctional dressing for infected wound management.	[84]
Chitosan–ethylenediaminetetraacetic acid (CS–EDTA) and Polyvinyl alcohol (PVA)	Lysozyme (LZ)	Electrospinning	Average fiber diameter was found to be 143–209 nm	The lysozyme-loaded chitosan–EDTA/PVA electrospun nanofibers exhibited rapid enzyme release, maintained antibacterial lytic activity, and significantly accelerated wound healing in vivo compared to conventional gauze dressings. Their smooth nanoscale morphology and incorporation of bioactive enzymes make them a promising therapeutic platform for enhanced wound repair and infection control.	[85]
Chitosan/Collagen/Poly(ethylene oxide) (PEO)	Curcumin	Single-step electrospinning	The nanofibers average range was between 112 and 196 nm.	The curcumin-loaded chitosan–collagen electrospun nanofibrous mats demonstrated excellent porosity, sustained antioxidant release, and enhanced cell adhesion and proliferation, making them highly effective for promoting wound repair. The synergistic combination of chitosan, collagen, and curcumin provided improved antibacterial, anti-inflammatory, and tissue-supportive properties, highlighting their potential as advanced bioactive wound-healing patches.	[86]
Chitosan	Cinnamaldehyde and silver nanoparticles (AgNPs)	Forcespinning (centrifugal spinning technology	Fiber diameter range 800–1500 nm	The chitosan-based composite fine fibers loaded with silver nanoparticles and cinnamaldehyde exhibited strong antibacterial activity against *Staphylococcus aureus* while maintaining excellent cytocompatibility and cell-supportive properties. Their non-toxic, three-dimensional fibrous architecture highlights their promising potential as multifunctional scaffolds for wound healing and tissue regeneration.	[87]
Chitosan (LPCS or HPCS)/Polyvinyl alcohol (PVA)/Polyethylene oxide (PEO)	Chitosan	Electrostatic spinning	226–423 nm	The *Periplaneta americana*-derived chitosan nanofibers demonstrated excellent mechanical strength, antibacterial activity, and biocompatibility, significantly enhancing wound closure, epithelialization, and collagen deposition in infected wounds. This study highlights a sustainable and cost-effective strategy for converting biological waste into valuable bioactive wound dressings with strong regenerative potential.	[88]
Poly(L-lactic acid) (PLLA), Poly(D-lactic acid) (PDLA), Quaternized chitosan (QCS), and PDLA-grafted QCS (QCS-PDLA)	Quaternized chitosan (QCS)	Electrospinning	300–800 nm	The stereocomplex poly(lactic acid)/chitosan derivative nanofibrous mats demonstrated enhanced thermal stability, mechanical strength, and multifunctional antibacterial and antioxidant properties, making them highly effective for infected wound management. Their ability to achieve complete wound closure within 15 days highlights their strong potential as advanced disinfectant wound dressings for full-thickness skin regeneration.	[89]
Polyvinyl alcohol (PVA)/Chitosan (CS)	Sulfanilamide/Silve nanoparticles	Electrospinning	Average nanofiber diameter was found to be 150 nm	The silver nanoparticle-decorated chitosan/PVA electrospun nanofibers loaded with sulfanilamide exhibited synergistic antibacterial activity and enhanced wound healing performance, demonstrating their effectiveness in infection control and tissue regeneration. The incorporation of in situ-synthesized silver nanoparticles further improved the therapeutic potential, making the composite nanofibers a promising multifunctional wound dressing system.	[90]
Chitosan/Polyvinyl alcohol	Ampicillin	Electrospinning	-----	The chitosan/PVA composite electrospun nanofibers exhibited an optimized biomimetic fibrous architecture at a 50:50 ratio, closely resembling the natural extracellular matrix and supporting their application in skin tissue regeneration. Combined with antibiotic loading and the inherent antibacterial properties of chitosan, these nanofibers represent a promising scaffold for infection-associated wound healing.	[91]
Polyvinyl alcohol/Chitosan	Tetracycline hydrochloride	Electrospinning	Fiber diameter range was 85–380 nm, depending on composition and crosslinking	The tetracycline-loaded PVA/chitosan electrospun nanofibrous mats demonstrated effective burst drug release, strong antibacterial activity against both Gram-positive and Gram-negative bacteria, and excellent cytocompatibility, making them highly suitable for infection-controlled wound healing. Their extracellular matrix-mimicking fibrous structure and ability to promote cell migration further highlight their potential as advanced antibacterial wound dressings.	[92]
Chitosan, Pullulan, combined with Chondroitin sulfate or Hyaluronic acid	Silver nanoparticles (AgNPs)	Electrospinning	Fiber diameter was found to be 500 nm (535–566 nm)	The silver nanoparticle-loaded chitosan/pullulan-based electrospun scaffolds demonstrated excellent antimicrobial activity, controlled biodegradation, and enhanced fibroblast proliferation, making them highly effective for chronic wound management. Among them, the chitosan/chondroitin sulfate scaffold showed superior regenerative potential by combining infection prevention with improved cellular growth, highlighting its promise as an advanced wound healing platform.	[93]

## Data Availability

No new data were created or analysed in this study. Data sharing is not applicable to this article.

## Abbreviations

The following abbreviations are used in this manuscript:

ECM	Extracellular Matrix
EGF	Epidermal Growth Factor
FGF	Fibroblast Growth Factor
HIF-1α	Hypoxia-Inducible Factor-1 Alpha
IL	Interleukin
MAPK	Mitogen-Activated Protein Kinase
MMPs	Matrix Metalloproteinases
NF-κB	Nuclear Factor Kappa B
Nrf2	Nuclear Factor Erythroid 2-Related Factor 2
PDGF	Platelet-Derived Growth Factor
PI3K	Phosphoinositide 3-Kinase
ROS	Reactive Oxygen Species
SPMs	Specialized Pro-Resolving Mediators
TGF-β	Transforming Growth Factor Beta
TIMPs	Tissue Inhibitors of Metalloproteinases
TLR	Toll-Like Receptor
TNF-α	Tumor Necrosis Factor Alpha
VEGF	Vascular Endothelial Growth Factor
VEGFR-2	Vascular Endothelial Growth Factor Receptor-2
